# Mucosal-Associated Invariant T-Cells: New Players in Anti-Bacterial Immunity

**DOI:** 10.3389/fimmu.2014.00450

**Published:** 2014-10-08

**Authors:** James E. Ussher, Paul Klenerman, Chris B. Willberg

**Affiliations:** ^1^Peter Medawar Building for Pathogen Research, University of Oxford, Oxford, UK; ^2^Department of Microbiology and Immunology, University of Otago, Dunedin, New Zealand; ^3^Oxford NIHR Biomedical Research Centre, John Radcliffe Hospital, Oxford, UK

**Keywords:** MAIT cells, anti-bacterial, inflammation, CD161, TCR, MR1

## Abstract

Mucosal-associated invariant T (MAIT) cells are an innate-like T-cell population involved in anti-bacterial immunity. In human beings, MAIT cells are abundant, comprising ~10% of the CD8^+^ T-cell compartment in blood. They are enriched at mucosal sites and are particularly prevalent within the liver. MAIT cells are defined by the expression of a semi-invariant T-cell receptor (Vα7.2-Jα33/12/20) and are restricted by the non-polymorphic, highly evolutionarily conserved MHC class Ib molecule, MHC-related protein (MR)1. MR1 has recently been shown to present an unstable pyrimidine intermediate derived from a biosynthetic precursor of riboflavin; riboflavin biosynthesis occurs in many bacteria but not in human beings. Consistent with this, MAIT cells are responsive to riboflavin-metabolizing bacteria, including *Salmonella*. In mouse models, MAIT cells have been shown to play a non-redundant role in anti-bacterial immunity, including against *Escherichia coli*, *Klebsiella pneumoniae*, and *Mycobacterium bovis* BCG. In human beings, MAIT cells are decreased in frequency in the blood of patients with tuberculosis or pneumonia, and their frequency has been inversely correlated with the risk of subsequent systemic bacterial infection in patients in intensive care. Intriguingly, MAIT cells are also depleted from the blood early in HIV infection and fail to recover with anti-retroviral therapy, which may contribute to the susceptibility of patients infected with HIV to certain bacterial infections, including non-typhoidal *Salmonella*. In this review, we will discuss what is currently known about MAIT cells, the role that *Salmonella* has played in elucidating MAIT cell restriction and function, and the role MAIT cells might play in the control of *Salmonella* infection.

## Introduction

**Figure 1:**
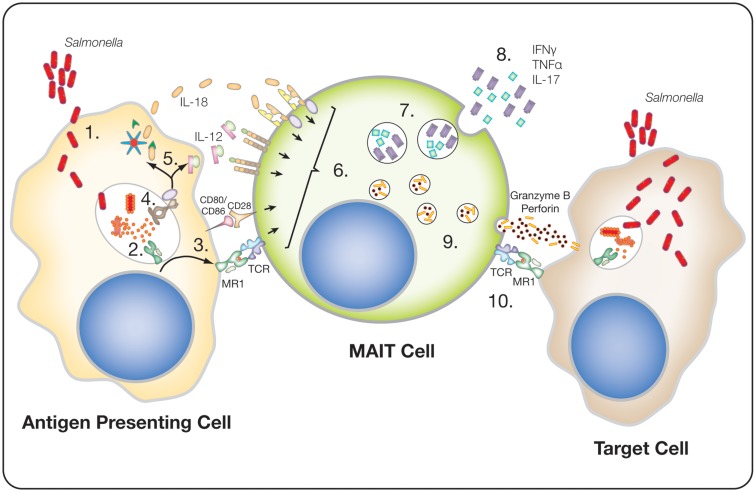
**Potential MAIT cell response to *Salmonella* infected cells**. **(1)** Internalization of *Salmonella* by an antigen-presenting cell, either through infection or actively by phagocytosis. **(2)** Lysis of the bacteria, within endocytic compartments, releases 5-A-RU, which is converted to 5-OE-RU or 5-OP-RU and binds to and stabilizes MR1. **(3)** The stable MR1 translocates to the cell surface, where it is presented along with other co-stimulatory molecules, e.g., CD80 or CD86. **(4)** Bacterial components trigger pathogen recognition receptors (PRR), such as TLR8. **(5)** PRR triggering drives cytokine expression, such as IL-12, and the activation of the inflammosome, resulting in the release of active-IL-18. **(6)** MAIT cells are activated either by TCR recognition of MR1 in combination with co-stimulatory receptors, e.g., CD28, and/or by cytokines, e.g., IL-12 and IL18. **(7)** Activated MAIT cells express pro-inflammatory cytokines, e.g., IFNγ, TNFα, and IL-17. **(8)** These cytokines can directly act anti-bacterially, or recruit and stimulate other immune cells, e.g., neutrophils by IL-17. **(9)** Activation of MAIT cells upregulates perforin and granzyme B expression. **(10)** Theoretically, the degranulation of cytotoxic granules into infected cells (target cells), via recognition of MR1, could induce cell death and, thus, the potential clearance of infected cells.

In 1999, Tilloy et al. first described mucosal-associated invariant T (MAIT) cells (1). Interest in this unique subset of innate-like T-cells has increased rapidly over the last 5 years as novel findings have revealed their unique anti-bacterial function and phenotype (2, 3). MAIT cells represent the most abundant innate-like T-cell population within human beings, comprising up to ~5% of the total T-cell population; this compares with just ~0.1% for invariant natural killer T (iNKT) cells (4, 5). They are characterized by the expression of a semi-invariant TCR (Vα7.2-Jα33/12/20) that recognizes the evolutionarily conserved MHC-like protein 1 (MR1), which presents a bacterial-derived ligand (6–11). Although they can be activated through their TCR, they are also readily stimulated by innate cytokines, either leading to the expression of pro-inflammatory cytokines or the release of cytotoxic and pro-inflammatory granzymes (Figure 1) (12, 13). Furthermore, MAIT cells have been associated with a number of disease settings, including bacterial infections (14), and pro-inflammatory diseases such as multiple sclerosis (15) and psoriasis (16). Thus, this large T lymphocyte population is likely to have an important role in human health.

This review will explore what is currently known about MAIT cells in human beings. Comparisons between human and murine MAIT cells have been made elsewhere (4). Furthermore, we will discuss the role that *Salmonella* has played in identifying the functions of this cell type, and the potential role MAIT cells may have in controlling *Salmonella* infections.

## MAIT Cell Phenotype

In addition to possessing the Vα7.2-Jα33/12/20 TCR, MAIT cells can be identified in human beings by the expression of a characteristic phenotypic signature composed of a number of additional surface and transcriptional markers.

### Memory phenotype

In adults, MAIT cells typically express an effector memory phenotype: CD45RO^+^, CCR7^−^, CD62L^−^, CD27^+^, and CD28^+^ (17–19). However, in cord blood, MAIT cells possess a naïve phenotype (CD45RA^+^, CCR7^+^, CD62L^+^), but still retain a phenotypic signature characteristic of adult MAIT cells, including the expression of CD161, interleukin (IL)-18Rα, CD8αα, and CCR6 (3, 5, 17, 20). A recent study demonstrated that MAIT cells in the thymus, spleen, and mesenteric lymph nodes of aborted second trimester fetuses also had a naïve phenotype and expressed only low levels of the characteristic MAIT cell markers, such as IL-18Rα and CD8αα, while MAIT cells in the fetal intestine, liver, and lung had a more memory phenotype (21).

### CD161

CD161 is a C-type lectin-like receptor originally identified by Lanier et al. (22). It is found on a broad range of lymphocytes, including CD4^+^, CD8^+^, γδ^+^ T-cells, and NK cells. The majority of NK cells express CD161 (>90%), while in the CD4^+^, CD8^+^, and γδ^+^ T-cell subsets, CD161 expression is limited to ~30% of cells (19, 23). However, within the CD8^+^ and CD8^−^ CD4^−^ T-cell population, CD161 expression can distinguish three separate subsets, CD161^−^, CD161^intermediate/+^, and CD161^high/++^; MAIT cells populate the CD161^++^ subset (17, 18). In adult peripheral blood, MAIT cells represent ~85% of the CD161^++^ subset (24). However, in cord blood, the MAIT cells make up a much smaller proportion of this subset, averaging ~15% of the CD161^++^ CD8^+^ T-cell population (21, 25, 26). During early childhood, this population expands so that by the age of 24 months the MAIT cell population already represents ~50% of the CD161^++^ CD8^+^ T-cell population (25).

The function of CD161 on MAIT cells is yet to be fully elucidated. On NK cells, binding of CD161 to its ligand [lectin-like transcript (LLT) 1] leads to an inhibition of cytotoxicity (27–29). Two studies explored the role of CD161 on CD8^+^ T-cells and reached opposing conclusions (27, 29). Rosen et al. found that cross-linking CD161 had no effect on anti-CD3/CD28 stimulated CD8^+^T-cells in terms of IFNγ expression and inhibited TNFα expression, whereas Aldemir demonstrated increased IFNγ expression after CD161 signaling. Le Bourhis et al. recently reported that ligation of CD161 on MAIT cells inhibited cytokine production but had no effect on cytotoxicity (30). One explanation for these differences could be due to the different CD161 cross-linking antibody clones used.

CD161 surface expression has recently been reported to be downregulated after long-term activation *in vitro*; downregulation was associated with greater rounds of proliferation (13). In HIV infection, loss of expression of CD161 by MAIT cells has been suggested (31). Given that MR1-tetramer-positive cells are not found in the CD161-negative population in healthy individuals (8), this requires further investigation.

### IL-18Rα

CD161 expression is associated with high levels of IL-18Rα expression. Of all human T lymphocytes, MAIT cells express the highest level of IL-18Rα (12, 17). Naïve MAIT cells, derived from cord blood, have also been shown to be IL-18Rα^+^ (5, 24). Recent observations of tissue from second trimester fetuses suggest that IL18Ra expression is low upon egress from the thymus but increases as MAIT cells mature in the tissues (21). Expression of IL-18Rα conveys an ability to respond robustly to cytokine stimulation (discussed below), which is limited in the other human T-cell populations (12).

### ZBTB16

The development of MAIT cells parallels that of NKT cells. Both innate-like T-cell subsets are selected for by non-classical MHC molecules, MR1 and CD1d, respectively, expressed on double negative thymocytes (32), and both subsets also express the transcription factor ZBTB16 (33). However, while NKT cells exit the thymus as an expanded and mature population, MAIT cells do not. Instead, murine models have shown that MAIT cells within the periphery have a naïve and immature phenotype, lacking expression of ZBTB16, CD25, CD69, or ICOS and further require an established microbiota and B-cells to expand into a memory phenotype (33). In contrast, MAIT cells found in the periphery of human fetuses have already acquired a more mature, memory phenotype, expressing ZBTB16, IL-18Rα and, upon bacterial stimulation, the capacity to express IFNγ and, in cells from the small intestines, IL-22 (5, 21). What drives selection and maturation *in utero* is unclear, but suggests that an endogenous ligand for MR1 may exist. Alternatively, this may reflect *in utero* exposure to commensal microflora, as has recently been suggested (34–36).

### RORγt

CD161 expression is also a key phenotypic maker of IL-17 secreting cells (19, 37). Within the CD4^+^T-cell subset, Th17 cells represent a novel helper cell population that can secrete IL-17A under the control of the master transcription factor retinoic acid-related orphan receptor (ROR) γt (38, 39). Within the CD8^+^T-cell subset, the expression of RORγt and the secretion of IL-17 are restricted to Tc17 cells that are CD161^++^. Thus, MAIT cells represent the vast majority of Tc17 cells (17, 24). The ability of MAIT cells to express IL-17A has recently been shown to depend on their pre-exposure to cytokines IL-1beta, IL-23, and IL-7 (40). Interestingly, hepatic stromal cells constitutively express IL-7, emphasizing the link between MAIT cells and the liver (41, 42).

### Co-receptor usage

CD161^++^/MAIT cells also encompass the CD8alpha/alpha population in adult blood, small bowel, and liver (5). Interestingly, this population is not seen in cord blood, or fetal thymus, but is seen at low levels in fetal intestine, liver, and lung (21), suggesting that it is derived from the CD8alpha/beta population through the regulation of CD8beta. Functionally and phenotypically, there is no difference between the CD8alpha/alpha and CD8alpha/beta subsets of the CD161^++^/MAIT cell population (5). However, Walker et al. have also described the expression of CD8alpha/alpha as the marker of terminally differentiated CD8^+^ T-cells that can be found in a number of chronic viral infections (26).

### Multidrug resistance transporter ABCB1

Mucosal-associated invariant T-cells express the ATP-binding cassette (ABC) B1 drug resistance transporter, which can also be seen on CD161^+^T-cells to a more limited degree (43). The ability to efflux drugs has been shown to allow MAIT cells to persist during chemotherapy for the treatment of acute myeloid leukemia (AML) or breast cancer (24, 43). The expression of the ABCB1 transporter possibly reflects the diverse environmental toxins MAIT cells are exposed to in their niche, although its overall role is not defined.

### Chemokine receptor expression

Mucosal-associated invariant T-cells express a range of chemokine receptors (CCR6, CCR5, CCR9, and CXCR6) that localize them to the gut, but more prominently to the lungs and liver (24, 44). The liver receives 25% of its blood supply from the hepatic artery and 75% from the portal vein. The portal vein delivers blood direct from the gastrointestinal track and spleen, placing the liver in the front line in the defense against microbial infection. Moreover, Balmer et al. recently described the liver as a firewall against infection from commensal bacteria that have invaded the body through either the intestines or systemic vascular circuits (45). Therefore, given their anti-bacterial function, it is unsurprising that MAIT cells represent up to 45% of all liver lymphocytes (24, 40). Furthermore, both CD161^+^ CD4^+^ and CD161^+^CD8^+^ T-cells are selectively recruited to the liver during inflammation (17, 23, 40).

## Anti-Bacterial Activity of MAIT Cells

Two studies in 2010, by Gold et al. and Le Bourhis et al., observed that MAIT cells could recognize antigens derived from a range of bacteria presented on antigen presenting cells (APCs) (2, 25). Gold et al. observed that a large population of CD8^+^ T-cells able to respond to *Mycobacterium tuberculosis* (Mtb) was present even in unexposed individuals. Functional and phenotypic analysis showed that this population was MR1 restricted and expressed the TCR Vα7.2 chain, i.e., MAIT cells. Furthermore, they showed that MAIT cells responded to *Salmonella enterica* serovar Typhimurium-infected dendritic cells, as well as *Escherichia coli* and *Staphylococcus aureus*. Le Bourhis et al. demonstrated the ability of MAIT cells to recognize and be activated by monocytes exposed to *E. coli* in an MR1-dependent manner. Furthermore, they observed that MAIT cells are activated by a wide range of bacteria (*E. coli, Klebsiella pneumoniae, Pseudomonas aeruginosa, Lactobacillus acidophilus, Staphylococcus aureus, Staphylococcus epidermidis*, and *Mycobacterium abscessus*) but not by all (*Enterococcus faecalis, Streptococcus pyogenes*). In addition, they showed activation in response to some yeasts (*Candida albicans, Candida glabrata*, and *Sacchromyces cerevisiae*). Moreover, MAIT cells were not observed in germ-free mice or in germ-free mice repopulated with *E. faecalis*, but were in germ-free mice repopulated with *Enterobacter cloacae* or *L. acidophilus*, underscoring the important role of certain bacterial species in MAIT cell development.

The ability of MAIT cells to control bacterial infections was elegantly demonstrated by Georgel et al. through the use of luminescent-*K. pneumoniae* (46). Despite the low frequency of MAIT cells in common laboratory strains of mice, they showed that in the absence of MR1, and, therefore, MAIT cells, mice succumbed to disseminated infection, while wild-type mice cleared the infection within 2 days. No defect in clearance was seen with *E. coli*, *Shigella dysenteriae*, or *Yersinia enterocolitica* suggesting that redundancy in the immune response is sufficient to control the dissemination of these organisms. In contrast, enhanced control of *E. coli* infection and *M. abscessus* infection was demonstrated in Vα19 or Vβ6 transgenic mice, which have an increased frequency of MAIT cells, on a MR1 replete background compared with a MR1^−/−^ background. Subsequent studies by Chua et al. and Meierovics et al. have demonstrated the need for MAIT cells in the early control of *Mycobacterium bovis*, BCG, and *Francisella tularensis* following a mucosal challenge (47, 48). Moreover, the presence of MAIT cells had a strong influence on the timing of recruitment and activation of conventional T-cells, and provided long-term protection alongside a conventional T-cell response (48).

## The MHC-Related Protein (MR)1 and its Ligand

The broad range of bacteria MAIT cells can respond to was recently explained with the identification of the ligand that binds MR1 (49). Kjer-Nielsen et al. discovered the structure of MR1 and the nature of the ligand that it binds after their serendipitous finding that 6-formyl pterin (6-FP), a photodegradation product of folic acid that was present in tissue culture media, was able to bind to MR1 and allowed its refolding. However, while 6-FP was able to stabilize the MR1 molecule, it was unable to activate MAIT cells. Given that the culture supernatant of *S. enterica* serovar Typhimurium was able to activate MAIT cells, they reasoned that it must contain the activating ligand. Therefore, they refolded MR1 with the culture supernatant of *S. enterica* serovar Typhimurium grown in minimal media that lacked vitamins (M9 minimal media), and analyzed the refolded MR1 by mass spectrometry. They observed a single ligand with a mass to charge ratio (*m/z*) of 329.11. This was consistent with the ligand being reduced 6-hydroxymethyl-8-d-ribityllumazine (rRL-6-CH2OH), a metabolic by-product of riboflavin metabolism. Chemical synthesis of reduced rRL-6-CH2OH confirmed that it had the same *m/z* as the ligand identified in the culture supernatant. Furthermore, synthetic reduced rRL-6-CH2OH, as well as related compounds derived from riboflavin metabolism, 7-hydroxy-6-methyl-8-d-ribityllumazine (RL-6-Me-7-OH) and 6,7-dimethyl-8-d-ribityllumazine (RL-6,7-diMe), were able to activate primary MAIT cells. This pivotal discovery was consistent with the prior observation that MAIT cells could be activated by *Salmonella*, along with other *Enterobacteriaceae*, *P. aeruginosa, S. aureus*, and some yeast species, which all contain the riboflavin synthetic pathway, but not by *S. pyogenes* or *E. faecalis*, which lack the riboflavin synthetic pathway (2, 25).

As the origin of the previously identified ligand, rRL-6-CH2OH, was not clear, Corbett et al. derived various strains of *Lactococcus lactis* with different mutations in the riboflavin synthesis operon and assessed their ability to activate MAIT cells (50). Mutants lacking genes involved in the riboflavin synthesis pathway (*ribA* and *ribG*) were unable to activate MAIT cells, and the MR1-binding ligand (*m/z* 329.11) was undetectable. By contrast, no defect in MAIT cell activation was seen with *ribB* and *ribH* mutants, pinpointing the production of 5-amino-6-d-ribitylaminouracil (5-A-RU), an early intermediate in riboflavin synthesis. The importance of 5-A-RU was confirmed by the lack of MAIT cell activation and absence of the MR1-binding ligand (*m/z* 329.11) in the culture supernatant of *S. enterica* serovar Typhimurium SL1344 with mutated *ribD* and *ribH* genes; furthermore, complementation restored reactivity. Despite the necessity for 5-A-RU, it was unable to bind MR1 or activate MAIT cells directly. However, upon non-enzymatic condensation with glyoxal or methylglyoxal, byproducts of other metabolic pathways (including glycolysis), 5-A-RU formed unstable intermediates [5-(2-oxoethylideneamino)6-d-ribitylaminouracil (5-OE-RU) and 5-(2-oxopropylideneamino)-6-d-ribitylaminouracil (5-OP-RU)], which were able to covalently bind to MR1. Furthermore, these unstable intermediates formed reversible covalent Schiff base complexes with Lys43, analogous to 6-FP (49). Moreover, 5-OE-RU and 5-OP-RU could be demonstrated in the culture supernatant of activating bacteria, but not of non-activating bacteria, and could be captured by MR1. Therefore, MR1 captures unstable intermediates that would otherwise convert to lumazines. MR1 refolded with 5-A-RU and methylgyoxal (5-OP-RU) produced a mass spectrometry peak of *m/z* 329.11, consistent with what was originally found in *Salmonella* supernatant (49). This suggested that 5-OP-RU, and not reduced rRL-6-CH2OH, was the true ligand for MR1. In support of this hypothesis, they also found a 315.09 *m/z* species (corresponding to 5-OE-RU) bound to MR1 refolded in the presence of *E. coli* DH5α; the 315.09 *m/z* species was also detected with *Salmonella*, but was much less abundant. This interesting finding demonstrates that different bacteria may produce different MAIT-activating ligands.

Overall, our current understanding of this complex process is that bacterially derived 5-A-RU is converted to 5-OE-RU or 5-OP-RU by reaction with bacterial or host-derived metabolites. These unstable intermediates covalently bind to, and stabilize MR1, which can then be recognized by the MAIT cell TCR.

Recently, Eckle et al. published that MAIT cells activation can be competitively inhibited by a novel MR1 ligand, acetyl-6-formylpterin (Ac-6-FP) (51). Ac-6-FP was identified in a search for analogs of 6-FP that were stronger antagonists of MAIT cell activation. MR1 efficiently refolded in the presence of Ac-6-FP and increased surface expression was seen in an MR1-transduced cell line treated with Ac-6-FP. Neither Ac-6-FP-MR1 tetramers nor 6-FP-MR1 tetramers were able to stain PBMCs. Consistent with antagonist activity, Ac-6-FP strongly inhibited the activation of the Jurkat.MAIT cell line in response to the agonist ligands, rRL-6-CH2OH and 5-OP-RU. Therefore, Ac-6-FP will be a useful tool in future studies of MAIT cell function.

## The MAIT Cell TCR

The defining feature of MAIT cells is the expression of a semi-invariant TCR, Vα7.2Jα33/12/20, which restricts MAIT cells to the evolutionarily conserved, non-polymorphic MHC-related protein 1 (MR1) (1, 6, 9, 11, 44). Vbeta chain usage does vary; however, it is dominated by Vβ2 and Vβ13.2 (1, 9, 11). Homologous TCRs have also been identified in mice (Vα19Jα33, paired with Vβ6 or Vβ8) as well as cattle (1, 5, 52).

The structure of the MAIT cell TCR and the residues that are critical for interacting with ligand-bound MR1 were determined prior to identification of the MAIT cell ligand (9, 11). Analysis of the MAIT cell TCR structure demonstrated that the CDR3α region is composed of the Vα7.2 and Jα33 of the alpha chain. *S. enterica* serovar Typhimurium was used as the source of the MR1-binding ligand to show individual residues in Vα7.2 (in CDR1α Gly28α and Asn30α, in CDR2α Tyr48α, and in CDR3α Asp92α, Asn94α, and Tyr95α), but not the Vβ chain, made critical contacts with MR1. However, switching the entire CDR3β loop with one from a non-MAIT cell TCR abolished recognition of MR1. A similar pattern was observed with other bacteria (*E. coli, K. pneumoniae, P. aeruginosa*, and *S. epidermidis*). Given the wide specificity, the authors suggested that the MAIT cell TCR was like a pattern recognition receptor. By mutational analysis, they identified residues Leu65 and Glu158 on MR1 as critical for the interaction with the TCR. These two residues mapped centrally to opposite sides of the antigen-binding cleft of MR1.

The identification of the ligand paved the way for the development of MR1 tetramers for the identification of MR1-restricted T-cells (8). Reantragoon et al. used MR1 with a K43A mutation to make tetramers. MR1 K43A could be refolded in the absence of ligand and once refolded was able to be loaded with reduced rRL-6-CH2OH. To further prove that the MR1-tetramer was specific to MAIT cells, PBMCs were depleted of MR1-tetramer binding cells and tested for reactivity to *S. enterica* serovar Typhimurium supernatant or to reduced rRL-6-CH2OH.

Using the loaded tetramers, Reantragoon et al. confirmed that the majority of MR1-restricted T-cells express the canonical semi-invariant T-cell receptor, Vα7.2-Jα33, but also identified minor (8–31%) MAIT cell populations exist that express Vα7.2-Jα12 and Vα7.2-Jα20. Interestingly, all alpha chains contained the conserved Tyr95α within CDR3α loop, which forms a hydrogen bond with RL-6-Me-7-OH bound by MR1 and is crucial for MAIT cell activation (8, 53). Interestingly, non-canonical TCRs showed a bias in Vβ usage toward TRBV6-4. Both canonical and non-canonical TCRs appeared to have the same ligand specificities. MR1-tetramer staining of lymphocytes isolated from the jejunal mucosa confirmed that MAIT cells are enriched at this site, with ~60% of CD3^+^ CD4-cells being MAIT cells. Furthermore, the majority of the tetramer positive cells in the jejunum were shown to be Vα7.2-Jα33, as in blood. This suggests that frequency of MAIT cells varies in different anatomical locations within the gastrointestinal tract, with higher frequencies in jejunum (8) than in ileum (54), colon, and rectum (31, 55). Alternatively, the variations observed in MAIT cell frequency in different tissues may reflect differences in methodology between studies.

Using a mouse MR1 tetramer in a MAIT-enriched mouse model (transgenic for Vα19 on Cα−/−background), the differences between mouse MAIT cells and human MAIT cells were highlighted (8). While human MAIT cells were predominantly CD8^+^ with some double negative cells and only a small CD4^+^ subset, in Vα19-transgenic mice >40% of MAIT cells were CD4^+^, with the remainder mostly DN rather than CD8^+^. Whether this reflects functional differences between human and mouse MAIT cells remains to be determined.

A recent study by Gold et al. investigated the TCR usage of MAIT cells that were responsive to different microbes (56). CD8^+^ T-cells from healthy donors were stimulated *ex vivo* with A549 cells infected with *Mycobacterium smegmatis, Salmonella typhimurium*, or *C. albicans*. Vα7.2 + CD8 + T-cells that produced TNFα were sorted and their TCR usage determined. They found greater diversity in the *TRAJ* gene usage than previously reported; while *TRAJ33* dominated, a range of other *TRAJ* genes were identified, including some (*TRAJ9* and *TRAJ39*) that do not encode for the Tyr95a residue that has been reported to be critical for MAIT cell activation (8, 11, 52). However, as the authors note, there is no allelic exclusion at the *TRA* locus, so the non-canonical *TRAJ* genes identified may not contribute to the functional MAIT cell TCR. *TRAJ* usage and CDR3α sequence of the responding MAIT cells differed between microbes; there was more similarity in the CDR3α sequence in MAIT cells activated by the same microbe than with those activated by a different microbe. Furthermore, there was significant diversity in *TRBV* gene sequences of MAIT cells responding to different microbes, although there was minimal overlap in the CDR3β sequence across donors and microbes. Overall, this suggested that different MAIT cells respond to different microbe-derived ligands. In support of this, of four MAIT cell clones that were robustly activated by *M. smegmatis*, only two were activated by RL-6,7-diMe. The authors speculate that MR1 ligand diversity drives MAIT cell TCR diversity and that that the Vα chain primarily mediates contact with MR1, while the CDR3β chain, positioned above the MR1 ligand-binding groove, contributes to ligand discrimination, as previously described by López-Sagaseta et al. (57, 58). This is consistent with the findings of Eckle et al., who showed that the novel inhibitory MR1 ligand, Ac-6-FP, induced structural alterations in MR1, which prevented the MAIT cell TCR CDR3β chain from binding (51). These two studies open up the exciting possibility that a wide range of novel MR1 ligands exists, which could modulate the MAIT cell response.

## MAIT Cell Activation

A number of studies have suggested adult MAIT cells to be terminally differentiated and, as a result, less responsive to TCR signaling, showing low IFNγ production and little proliferation compared to stimulation that bypasses the TCR (such as activation by PMA and ionomycin, or PHA) (24, 33, 43). In contrast, MAIT cells derived from cord blood or fetal tissues readily proliferate with TCR stimulation (21, 33). This lack of responsiveness by adult MAIT cells to TCR stimulation can, however, be overcome. Turtle et al. demonstrated that TCR signaling required the addition of an innate signal/s from either the co-receptor CD28 or the cytokine IL-12 in order to induce high levels of IFNγ and strong proliferation (20). Using transcriptional profiling, they went on to show that the mechanisms controlling TCR signaling in MAIT cells were distinct from those seen in anergic or exhausted T-cells.

A study by Chua et al. demonstrated that in a murine model of bacterial infection using *M. bovis* BCG, IL-12 signaling, but not TCR signaling, was required for the control of infection; blockade of IL-12 but not MR1, inhibited the anti-bacterial activity of MAIT cells (47). This regulation of activation is similar to that seen in iNKT cells, where IL-12 signaling dominates over CD1d-induced TCR signaling (59, 60). In human models of MAIT cell activation, there is a dual role for TCR- and cytokine-induced activation of MAIT cells. At early time points, 5 hours after a MAIT cell encounters an antigen-presenting cell (APC) presenting its cognate antigen on MR1, TCR signaling dominates activation (12). However, at later time points (20 hours), cytokine-mediated activation is equally important and MR1 blockade has a more limited effect. In contrast to the murine models, IL-12 alone is not sufficient to induce IFNγ expression, but requires the addition of other innate cytokines, such as IL-18 (12). The ability to respond to IL-12 plus IL-18 is similar to that of NK cells (61), and implicates MAIT cells in a range of infectious and non-infectious inflammatory diseases. Furthermore, signaling via toll-like receptors (TLR) is able to drive the expression of a range of pro-inflammatory cytokines from professional APCs, which can activate MAIT cells (Figure 1) (12, 62). We have shown that TLR8 agonists are particularly potent stimulators of IL-12 and IL-18 secretion, and therefore capable of driving MAIT cell IFNγ expression (12, 62). Thus, this suggests that in addition to their anti-bacterial role, MAIT cells may play an important role in anti-viral responses, and this may also provide a mechanism that explains their involvement in other pro-inflammatory settings such as experimental autoimmune encephalomyelitis, multiple sclerosis, inflammatory bowel disease, psoriasis, and arthritis (15, 16, 54, 63, 64).

## MAIT Cell Cytotoxicity

How MAIT cells affect their anti-bacterial function remains poorly defined. Upon activation, MAIT cells are able to produce several cytokines, including IFNγ, TNFα, and IL-17 (17, 24). In addition, it has recently been demonstrated that MAIT cells are also cytotoxic. Le Bourhis et al. reported that MAIT cells can recognize epithelial cells (HeLa cells) infected with *Shigella flexneri* but not *S. enterica* serovar Typhimurium (30). Consistent with this, MAIT cells were able to kill HeLa cells infected with *S. flexneri* but not *S. enterica* serovar Typhimurium. This cytotoxicity was dependent upon MR1 and was evident in HeLa cells expressing endogenous levels of MR1. No reactivity was seen with a *Salmonella* pathogenicity island 1 (SpI-1)-deleted strain of *S. enterica* serovar Typhimurium (SpI-1 is required for invasion). Similarly, MAIT cell activation and cytotoxicity was not seen with a SpI2-deleted strain of *S. enterica* serovar Typhimurium. Therefore, the virulence factors that prevent MR1 loading remain to be defined. The authors’ suggestion was that *Salmonella* avoids detection as it resides in vacuoles and prevents fusion with the lysosome, while *Shigella* escapes to the cytoplasm. Indeed, invasion by *Shigella* was important as HeLa cells infected with a DMxiD strain were unable to efficiently activate MAIT cells. *In vivo* activation (determined by HLA-DR expression) and decreased frequency of MAIT cells in blood was observed in human beings after oral challenge with an attenuated strain of *S. dysenteriae* suggesting that MAIT cells responded to *S. dysenteriae in vivo*.

Recently, Kurioka et al. described increased cytotoxic potential of MAIT cells after activation (13). These cytotoxic, or “licensed,” MAIT cells displayed upregulation of granzyme B, normally not expressed in resting MAIT cells, and enhanced perforin expression, which is low in resting MAIT cells (13). The licensed MAIT cells displayed an increased capacity to kill cognate-target cells, and maintained this phenotype even after several rounds of proliferation.

## A Role for MAIT Cells in the Control of *Salmonella* Infection

The role of MAIT cells in *Salmonella* infection remains to be defined. As discussed above, MAIT cells can be activated by *Salmonella* sp. *in vitro* (8, 11, 25). MAIT cells activated by *Salmonella* sp. produce IFNγ and TNFα; these cytokines have been shown to be important in the control of *Salmonella* infection (65). In addition, MAIT cells can secrete IL-17 in response to stimulation with *E. coli* (55). IL-17 has recently been suggested to be critical in preventing the dissemination of *Salmonella*. In IL-17 receptor-deficient mice, increased systemic dissemination of *S. enterica* serovar Typhimurium was seen (66). Furthermore, in SIV-infected rhesus macaques, increased dissemination of *S. enterica* serovar Typhimurium was seen, which was associated with loss of Th17 cells and the IL-17 response from the ileum (66). Therefore, along with the loss of Th17 cells (67), the loss of MAIT cells in HIV infection may contribute to the increased susceptibility to disseminated non-typhoidal *Salmonella* infection (68).

Mucosal-associated invariant T-cells may also contribute to the control of *Salmonella* infection through cytotoxic activity. Cytotoxic T-cells are important in the clearance of *Salmonella* infection (69). However, MAIT cells are unable to kill HeLa cells, an epithelial cell line, infected with *Salmonella* (69). This may be because live *Salmonella* sp. is able to avoid MR1 containing compartments by preventing phagosome-lysome fusion (70). Future investigations with live *Salmonella* sp. may help elucidate the MR1 loading pathway/s, and subsequent activation of MAIT, revealing potential therapeutic targets.

The low numbers of MAIT cells in common laboratory mouse strains has hampered the study of the role MAIT cells in response to *Salmonella* infection. Therefore, a robust murine model is required to investigate the role of MAIT cells in the control of *Salmonella* infection.

## MAIT Cells in Infectious Disease

The role of MAIT cells in human disease has not been fully assessed due to the difficulties of obtaining tissue samples. However, there are a number of interesting associations between the frequency of MAIT cells within peripheral blood and disease. During active *M. tuberculosis* infection, MAIT cells numbers are lower in peripheral blood compared to healthy controls (2, 25, 71, 72). Consistent with these findings, Grimaldi et al. looked at MAIT cell numbers in critically ill septic and non-septic patients (14). They observed that all critically ill patients studied, including those with severe bacterial or viral infections and those with non-infective illness, had low MAIT cell counts compared to healthy controls. This was least pronounced in individuals with severe viral infections, and most striking in individuals infected with bacteria other than *Streptococcus* species (14). This suggests that the loss of MAIT cells from the periphery could be due, in part, to compartmentalization during disease (2, 25). Interestingly, however, the authors also observed that those individuals with persistent MAIT cell depletion at day 4 post-admission were at increased risk for subsequent nosocomial infections. Therefore, MAIT cell exhaustion or death, two mechanisms proposed to occur in HIV (see below), may contribute to this phenotype.

In both HCV and HIV, MAIT cells are depleted from the blood (17, 31, 55). During HCV infection, the loss of peripheral MAIT cells is potentially due to their relocation to the liver (17). Moreover, the frequency of IFNγ and IL-17 dual-expressing CD8^+^ T-cells in the liver, a proxy for MAIT cells, was inversely correlated with the fibrosis score, suggesting that they either play a protective role during HCV infections, or that they are progressively lost from the liver with increasing fibrosis. This might contribute to the higher rates of bacteremia seen in individuals with cirrhosis (45, 73, 74).

In HIV infection, the loss of CD161^++^ MAIT cells from blood occurs early during infection and persists despite otherwise successful anti-retroviral therapy (31, 55, 71, 75, 76), although the nature of this perturbation is unclear. Cosgrove et al. reported that MAIT cells, defined as CD161^++^ CD8^+^ T-cells by flow cytometry, were depleted as a proportion of the CD8^+^ T-cell population in blood (55). They proposed that this depletion was due, at least in part, to activation induced cell death from overstimulation secondary to microbial translocation. While Leeansyah et al. also observed a decrease in size of the CD161^++^Vα7.2^+^ population, they suggested that this was due to downregulation of CD161 and functional exhaustion of MAIT cells (31). In support of this, they noted an increase in frequency of CD161-Vα7.2^+^ T-cells as a proportion of CD3^+^ T-cells. Importantly, the antibody against Vα7.2 used in these studies is not specific for the canonical MAIT cell TCR (33). Therefore, the recently described MR1 tetramer will be useful to determine whether the CD161-Vα7.2^+^ T-cells seen in HIV infection are MAIT cells or not (8). Interestingly, in healthy donors, MR1 tetramer does not bind the CD161-Vα7.2^+^ T-cell population.

## MAIT Cells in Inflammatory Disease

In addition to their anti-microbial activity, MAIT cells have also been implicated in a range of pro-inflammatory settings. Serriari et al. observed that individuals with inflammatory bowel diseases had lower numbers of circulating MAIT cells compared to controls (54), as seen during bacterial and viral infections (2, 17, 25, 54, 71). However, within individuals, the frequencies of MAIT cells were increased within inflamed tissues compared to healthy tissue, suggesting recruitment of MAIT cells from the blood to sites of inflammation. This is a theme consistent in other inflammatory diseases such as psoriasis, rheumatoid arthritis, multiple sclerosis, and experimental autoimmune encephalomyelitis (15–17, 63, 77). Interestingly, CD56^−^ MAIT cells have been observed infiltrating kidney and brain tumors, implying a potential role in tumor immunity (78).

Overall, these studies in human disease demonstrate that MAIT cells are a population of innate-like cells that rapidly translocate to sites of inflammation, regardless of whether the inflammation is due to bacterial infection or to other pro-inflammatory stimuli.

## What the Future Holds for MAIT Cell Research

Through the use of tools such as *Salmonella*, our understanding of MAIT cell functions has increased rapidly over the last 5 years. However, there are still a number of important questions to be answered. Are there other ligands presented by MR1? Is there an endogenous ligand within the thymus for MAIT cell selection? How is MR1 regulated? What are the relative roles of TCR-dependent and TCR-independent triggering of MAIT cells in host defense? Understanding what MR1 presents and how it is regulated will be critical for understanding where and when MAIT cells have a definitive role in disease. What role MAIT cells play during human disease, in both infectious and autoimmune settings also needs to be addressed. Although it is important to study human disease, much will be learnt from animal models. Understanding how MAIT cells are regulated will potentially allow their *in vivo* manipulation for a positive outcome. Given the rise of antibiotic resistant bacteria, as highlighted by the recent WHO report (April 2014) future prophylactic and therapeutic strategies that harness the anti-bacterial potential of MAIT cells may be particularly important (79, 80).

## Conflict of Interest Statement

The authors declare that the research was conducted in the absence of any commercial or financial relationships that could be construed as a potential conflict of interest.
